# Analysis of cuproptosis-related genes in Ulcerative colitis and immunological characterization based on machine learning

**DOI:** 10.3389/fmed.2023.1115500

**Published:** 2023-07-17

**Authors:** Zhengyan Wang, Ying Wang, Jing Yan, Yuchi Wei, Yinzhen Zhang, Xukai Wang, Xiangyang Leng

**Affiliations:** ^1^Changchun University of Chinese Medicine, Changchun, China; ^2^The Affiliated Hospital of Changchun University of Chinese Medicine, Changchun, China; ^3^Department of Orthopedics, The Affiliated Hospital of Changchun University of Chinese Medicine, Changchun, China

**Keywords:** cuproptosis, Ulcerative colitis (UC), machine learning, immune infiltration, cuproptosis-related genes (CRGs)

## Abstract

Cuproptosis is a novel form of cell death, mediated by protein lipid acylation and highly associated with mitochondrial metabolism, which is regulated in the cell. Ulcerative colitis (UC) is a chronic inflammatory bowel disease that recurs frequently, and its incidence is increasing worldwide every year. Currently, a growing number of studies have shown that cuproptosis-related genes (CRGs) play a crucial role in the development and progression of a variety of tumors. However, the regulatory role of CRGs in UC has not been fully elucidated. Firstly, we identified differentially expressed genes in UC, Likewise, CRGs expression profiles and immunological profiles were evaluated. Using 75 UC samples, we typed UC based on the expression profiles of CRGs, followed by correlative immune cell infiltration analysis. Using the weighted gene co-expression network analysis (WGCNA) methodology, the cluster’s differentially expressed genes (DEGs) were produced. Then, the performances of extreme gradient boosting models (XGB), support vector machine models (SVM), random forest models (RF), and generalized linear models (GLM) were constructed and predicted. Finally, the effectiveness of the best machine learning model was evaluated using five external datasets, receiver operating characteristic curve (ROC), the area under the curve of ROC (AUC), a calibration curve, a nomogram, and a decision curve analysis (DCA). A total of 13 CRGs were identified as significantly different in UC and control samples. Two subtypes were identified in UC based on CRGs expression profiles. Immune cell infiltration analysis of subtypes showed significant differences between immune cells of different subtypes. WGCNA results showed a total of 8 modules with significant differences between subtypes, with the turquoise module being the most specific. The machine learning results showed satisfactory performance of the XGB model (AUC = 0.981). Finally, the construction of the final 5-gene-based XGB model, validated by the calibration curve, nomogram, decision curve analysis, and five external datasets (GSE11223: AUC = 0.987; GSE38713: AUC = 0.815; GSE53306: AUC = 0.946; GSE94648: AUC = 0.809; GSE87466: AUC = 0.981), also proved to predict subtypes of UC with accuracy. Our research presents a trustworthy model that can predict the likelihood of developing UC and methodically outlines the complex relationship between CRGs and UC.

## Introduction

UC is a chronic and recurrent inflammatory bowel disease that begins in the rectal mucosa (1). Active superficial inflammation may extend proximally, accumulate, and spread to part of or the entire colon (2). Most people with UC are between the ages of 30 and 40. Typical clinical symptoms are frequent purulent stools, abdominal pain and diarrhea, urinary urgency, fatigue, and weight loss (3, 4). UC can be caused by a variety of factors, such as genetic susceptibility and stimulation by environmental triggers, but the exact etiology and pathogenesis are not known (5, 6). There has been a steady rise in the number of UC patients worldwide in recent years, resulting in a serious socio-economic burden. Therefore, we need to further investigate potential new therapeutic targets to predict the development of the disease.

**Figure 1:**
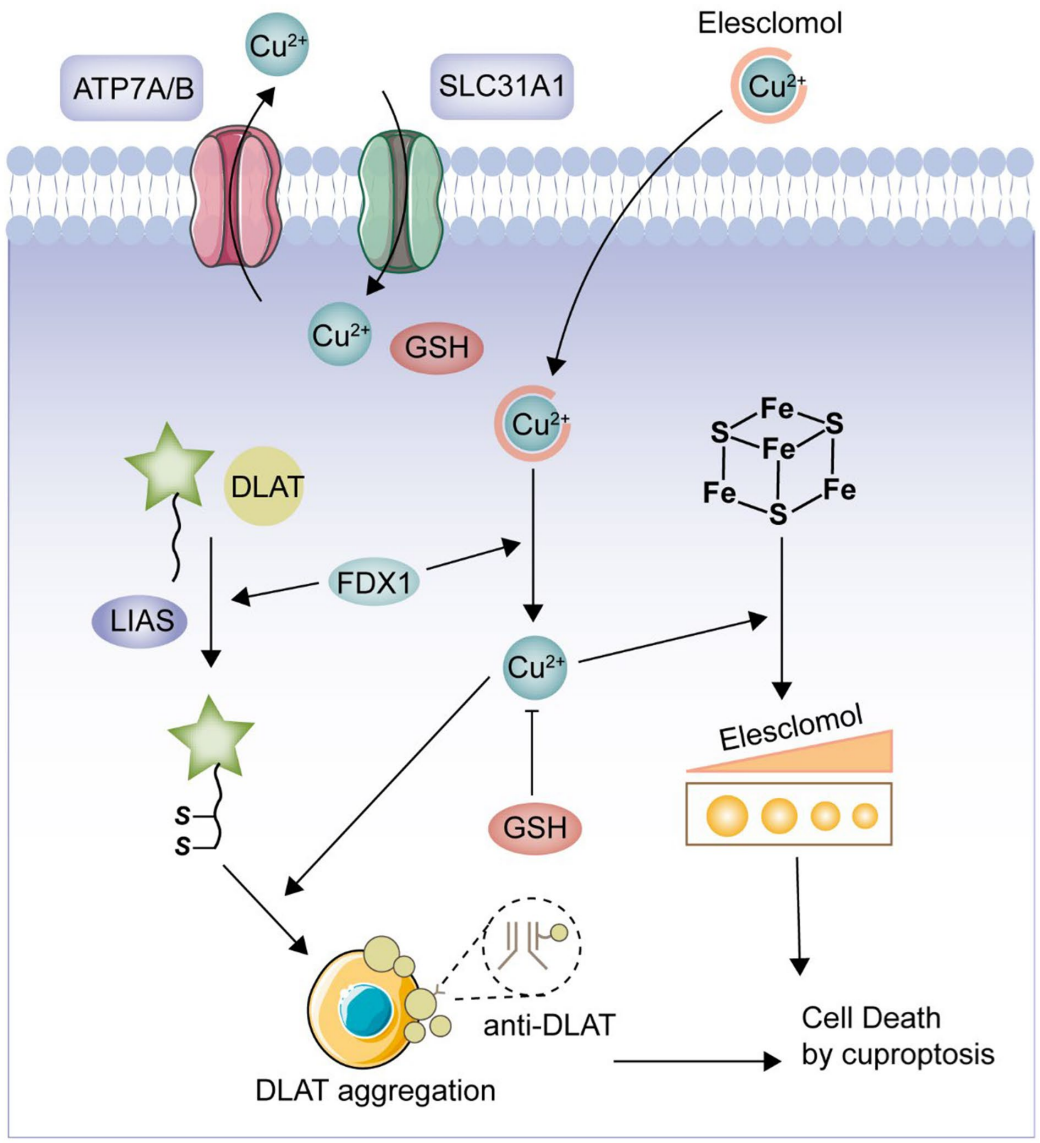
Cuproptosis pathway. FDX1 plays a crucial role as a copper ion carrier in the induction of cell death and is involved in the regulation of protein lipoylation. Elevated levels of copper promote the aggregation and functional impairment of lipoylated proteins, leading to instability of iron–sulfur cluster proteins, protein toxicity stress, and ultimately cell death. Moreover, excessive copper can bind to lipoylated DLAT, triggering abnormal oligomerization of DLAT and the formation of DLAT foci. This process contributes to cellular protein toxicity stress, further exacerbating cell death.

Cuproptosis is a novel form of cell death, mediated by protein lipid acylation and highly associated with mitochondrial metabolism, which is regulated in the cell (7). Copper is both an essential cofactor and an essential micronutrient for all organisms, but in excess, it can lead to cell death (8). According to a prior study, the lipidated parts of the tricarboxylic acid (TCA) cycle serve as the direct sites of copper’s direct binding, which causes copper-dependent mortality (9). Excess copper leads to the aggregation of lipoylated dihydrolipoamide S-acetyltransferase (DLAT), which triggers proteotoxicity and ultimately cell death (10). This is a novel type of cell death, in contrast to prior studies that have described a variety of types of carefully controlled programmed cell death, including apoptosis, pyroptosis, necroptosis, and iron apoptosis (11). Currently, multiple studies have shown that CRGs play an important regulatory role in the development and progression of a variety of tumors (12). However, there are no bioinformatics-based studies such as machine learning to demonstrate the regulatory role of CRGs in UC. Therefore, in the present study, we intended to comprehensively investigate the relevant CRGs in UC and their clinical significance. We selected 19 CRGs for this study based on the published papers (NFE2L2, NLRP3, ATP7B, ATP7A, SLC31A1, FDX1, LIAS, LIPT1, LIPT2, DLD, DLAT, PDHA1, PDHB, MTF1, GLS, CDKN2A, DBT, GCSH, and DLST) (13–17). Furthermore, we constructed a pathway of cuproptosis based on previously published and comprehensive *in vivo* and *in vitro* experiments (Figure 1) (8, 9). Our analysis highlights the importance of CRGs in the development of UC and hopefully will provide a useful contribution to subsequent UC studies.

In the present study, we used the GSE107499 dataset to systematically investigate for the first time the expression differences and immune correlations of CRGs between normal and UC samples. Based on the expression of CRGs, we divided 75 UC samples into two clusters, and then we looked at the immune cell differences between the two clusters as well as the relationship between immune cells and CRGs. Subsequently, the most differentially expressed gene modules were chosen after the WGCNA algorithm had discovered particular DEGs, and based on cluster-specific DEGs, a richness of biological activities and pathways were discovered. Additionally, by contrasting various machine learning model methods, several machine learning models were constructed, and we selected the best predictive model for subsequent analysis. Finally, the accuracy of the prediction models was validated using nomograms, calibration curves, DCA, and five independent external datasets.

## Materials

### Data sources and processing

The Gene Expression Omnibus (GEO) database (18) is a sub-database of the National Center for Biotechnology Information (NCBI) and is used to store gene expression datasets. We downloaded six datasets from the GEO database, GSE107499 (Controls = 44, UC = 75), GSE11223 (Controls = 73, UC = 129), GSE38713 (Controls = 13, UC = 30), GSE53306 (Controls = 12, UC = 28), GSE94648 (Controls = 22, UC = 25), and GSE87466 (Controls = 21, UC = 87) (19–23). The GSE107499 dataset was used for the analysis of this study. Five data sets (GSE11223, GSE38713, GSE53306, GSE94648, and GSE87466) were used for independent validation. The raw gene expression data from the six GEO datasets were analyzed and standardized using the robust multiarray average method.

### Identification of DEGs

We used the GEO database’s GEO2R tool to screen and visualize differentially expressed genes using | log FC | ≥ 1.0 and adjusted *p* value <0.05 as screening criteria. The GEO2R function is implemented based on the “limma” R package. The “limma” R package is a generalized linear model-based differential expression screening method that can obtain DEGs between different comparison groups and controls (24). Specifically, we obtain the gene expression profile data set, remove the genes with an expression value greater than 50%, then use the “voom” function to transform the data, further using the “ImFit” function to perform multiple The data were then transformed using the “voom” function, and further multiple regression using the “ImFit” function was performed to further compute moderated t-statistics, moderated F-statistics, and log-odds of differential expression by empirical Bayes moderation of the standard errors toward a common value to finally obtain the significance of differences for each gene. The Metascape database (25) is a biological database that allows enrichment analysis online. The Metascape database was then used for Gene Ontology (GO) and Kyoto Encyclopedia of Genes and Genomes (KEGG) enrichment analysis and visualization of the differentially expressed genes in UC. Then, the correlation analysis and identification of DEGs in the CRGs of the GSE107499 dataset were performed using the “corrplot” R package based on Spearman’s statistical method and the “limma” R package.

### Evaluation of immune cell infiltration based on the CIBERSORT algorithm

CIBERSORT is an analytical tool for estimating gene expression profiles and using gene expression data to make relative estimates of the abundance of cell types in mixed cell populations. CIBERSORT is based on linear support vector regression (26). We estimated the relative abundance of 22 immune cell types in each sample of GSE107499 gene expression data using the CIBERSORT algorithm and the LM 22 feature matrix. For each sample, CIBERSORT calculates an inverse fold product *p* value using Monte Carlo sampling. Each sample’s 22 immune cell proportions added up to 1 in total. Only samples with a *p*-value of 0.05 or lower were regarded as precise immune cell fractions. The relationship between CRGs and immune cells linked with UC was next examined. Initially, using spearman statistical methods, correlation coefficients between the expression of CRGs and the relative fraction of immune cells were looked at. A *p* value less than 0.05 was then identified as a significant association using the Spearman correlation coefficient. At last, the “corrplot” R tool (version 0.92) was used to show the findings.

### Unsupervised clustering of UC patients

Based on data from associated copper death gene expression profiles, unsupervised cluster analysis (27) was carried out. Using a k-means algorithm with 1,000 iterations, the 75 UC samples were divided into various clusters. We determined the optimal number of clusters based on a combination of cumulative distribution function (CDF) curves, consistency matrices, and consistency clustering scores >0.8 and selected the maximum number of subtypes (*k* = 9) for analysis. Principal component analysis (PCA) (28), one of the most widely used algorithms for dimensionality reduction of data, was subsequently performed on the two clusters after clustering. Finally, the clustered groups were analyzed for differences and correlations in the CRGs.

### WGCNA and gene set variation analysis (GSVA) of clusters

With the aid of the analytical technique WGCNA (29), it is possible to analyze the gene expression profiles of several samples, classify genes with similar expression patterns, and look into the associations between certain traits or phenotypes and modules. Using the optimal soft threshold, a weighted proximity matrix was created, following which a topological overlap matrix was created. A unique color is assigned to every module. The module signature genes are a representation of the overall gene expression profile in each module. The importance of modules demonstrates the association between modules and illness states. GSVA in order to clarify the variations in the collection of enriched genes between various CRGs clusters. By contrasting the GSVA scores between the various CRGs clusters, many expression pathways and biological processes were discovered. Subsequently, the investigation of immune cell infiltration was then repeated for several CRGs clusters.

### Building machine learning predictive models

In recent years, more and more machine learning and deep learning methods have been widely used in the medical field with outstanding results. XGB, SVM, RF, GLM, and adaptive boosting (AdaBoost) are some of the most commonly used methods for machine learning, while artificial neural networks (ANN), multilayer perceptron (MLP), and fully neural network (FNN), often referred to simply as neural networks, are the foundation of deep learning. Machine learning favors the interpretability of the model, while deep learning is more concerned with the accuracy of the model. Machine learning is more applicable to tabular data with a relatively small number of variables, while deep learning methods are specifically designed for large data and large feature sets and are more applicable to images or other data with a large number of variables (30, 31). Both AdaBoost and XGB are built based on the boosting algorithm. AdaBoost locates the deficiencies of the model by boosting the weights of the error points, while XGB locates the deficiencies of the model by counting the gradient. Therefore, compared with AdaBoost, the XGB model can use more kinds of objective functions (32). In addition, the dataset selected for this study is from the GEO database, which has a small sample size. Therefore, four machine learning methods—XGB, SVM, RF, and GLM—are used for this experimental study. The four machine learning models XGB, SVM, RF, and GLM were built using the “caret” R package (version 6.0.91) to find differentially expressed genes shared by the UC and turquoise modules. XGB is a supervised model that enables thorough comparisons of classification error and model complexity. It is built on a set of gradient-enhanced augmented trees (33). A binary classification model called SVM transfers the feature vector of an instance to a set of spatial points. The optimum separating hyperplane that maximizes the positive and negative sample intervals on the training set will be found via SVM, as will the best partitioning hyperplane that can distinguish between two classes and maximize the interval (34). RF is an integrated machine learning technique that predicts classification or regression utilizing different independent decision trees by randomly selecting samples and attributes during node splitting (35). GLM, a development of the linear model, establishes the mathematical expectation of the response variable by linking a linear combination of predictor variables (36). The four machine learning models discussed above were interpreted using the “DALEX” package (version 2.4.0), which was also used to show the residual distribution and feature significance. The “pROC” R package (version 1.18.0) was used to visualize the ROC curve. In conclusion, the primary predictive genes associated with UC were found to be the top five significant variables, and the best machine learning models were found.

### Nomogram model construction and independent validation analysis

To determine the prevalence of UC, a nomogram model was developed. Each predictor is given a score, and the “total score” of all predictors is calculated by adding the individual scores of the predictors mentioned above. Using calibration curves and DCA, the Nomogram model’s prediction accuracy was calculated. In addition, five datasets (GSE11223, GSE38713, GSE53306, GSE94648, and GSE87466) were used for independent validation analyses. The ROC curves for these datasets were constructed and visualized using SPSS 25.0 for the prediction model, which was used to validate the accuracy of the prediction model in differentiating between non-UC and UC patients.

## Results

### Identification of DEGs and differential analysis of CRGs

**Figure 2:**
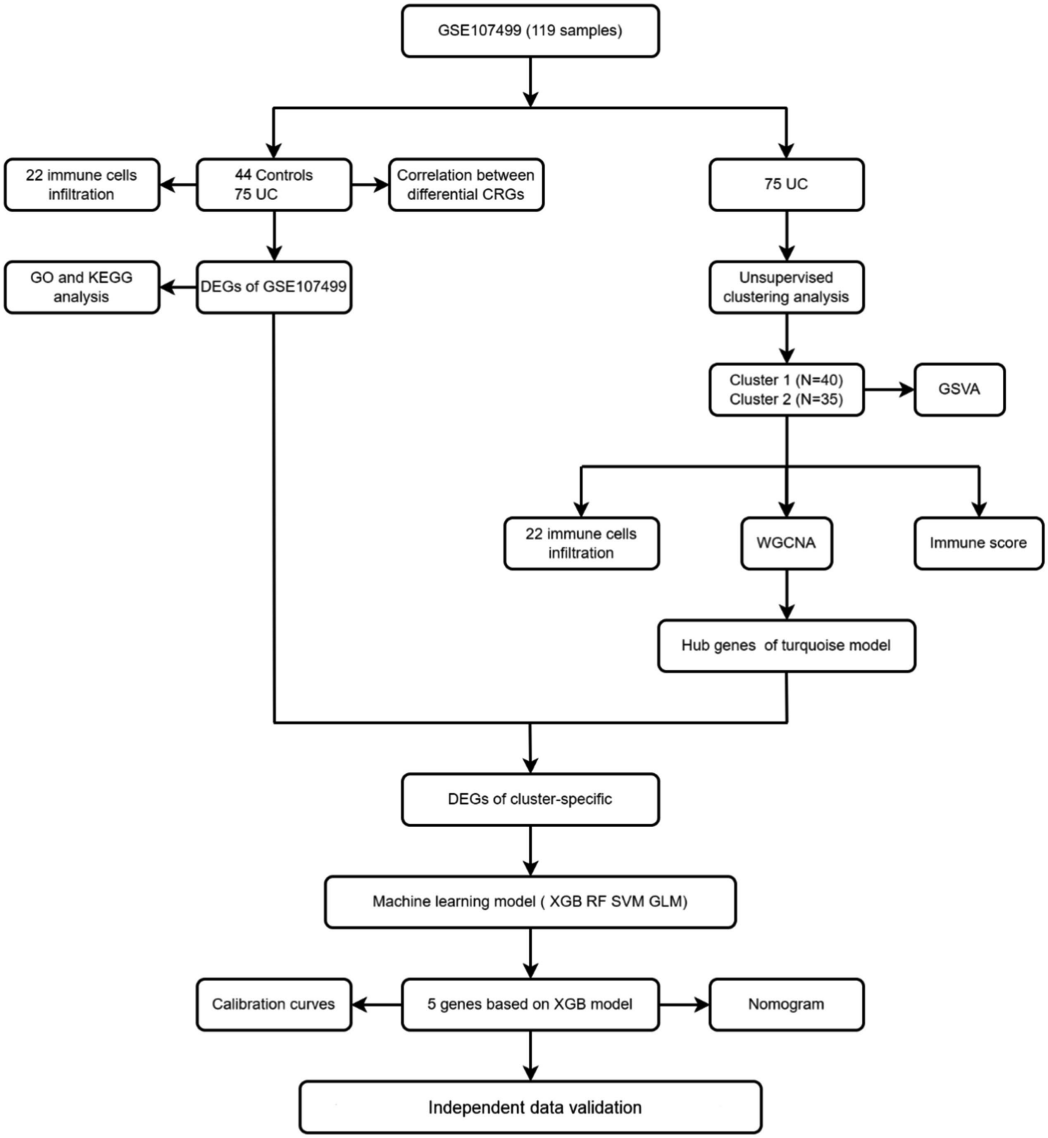
Flow chart.

**Figure 3:**
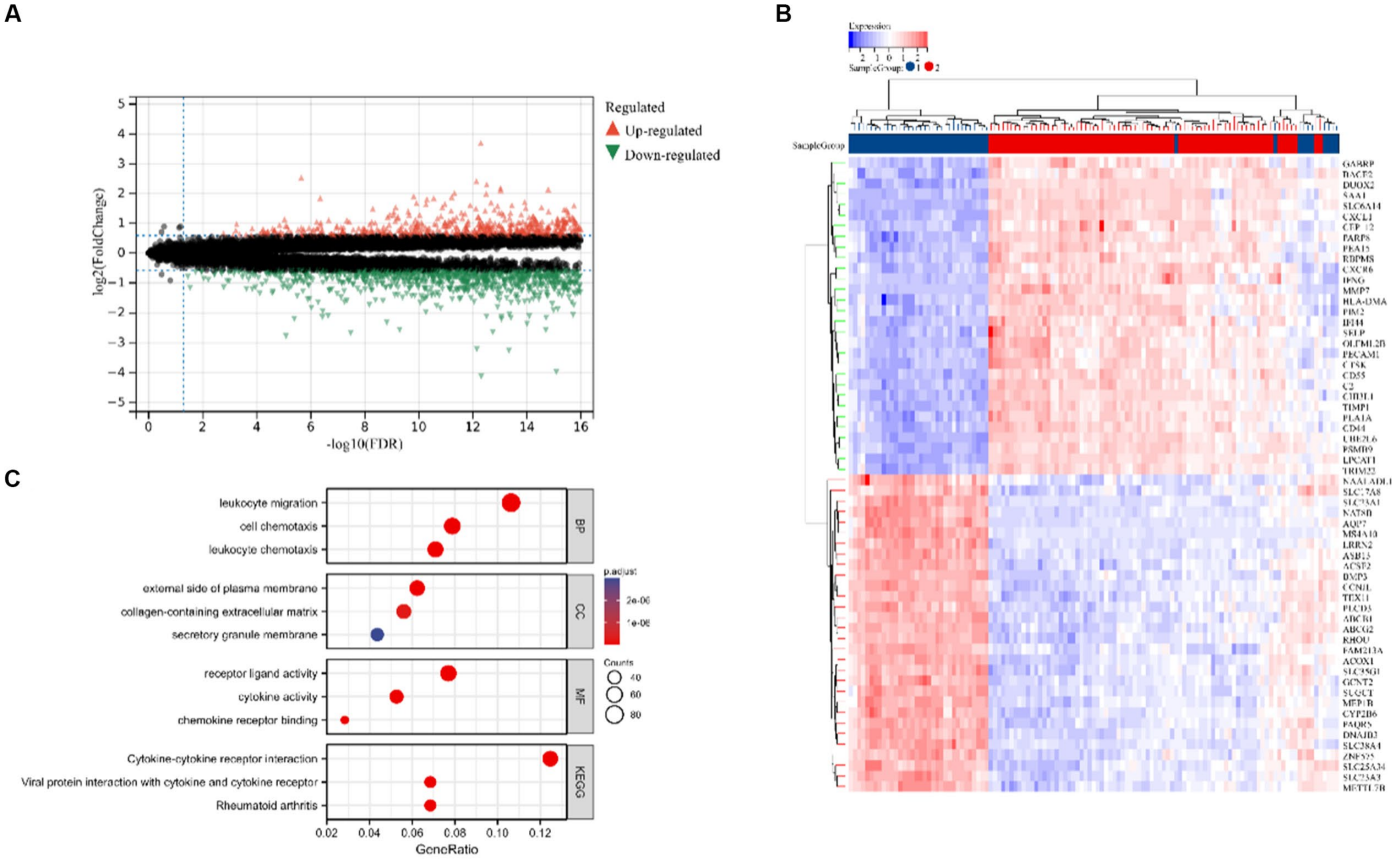
Identification of DEGs in UC. **(A)** Volcano map of DEGs. **(B)** The top 30 up-regulated and down-regulated genes of UC. **(C)** Analysis of GO and KEGG.

**Figure 4:**
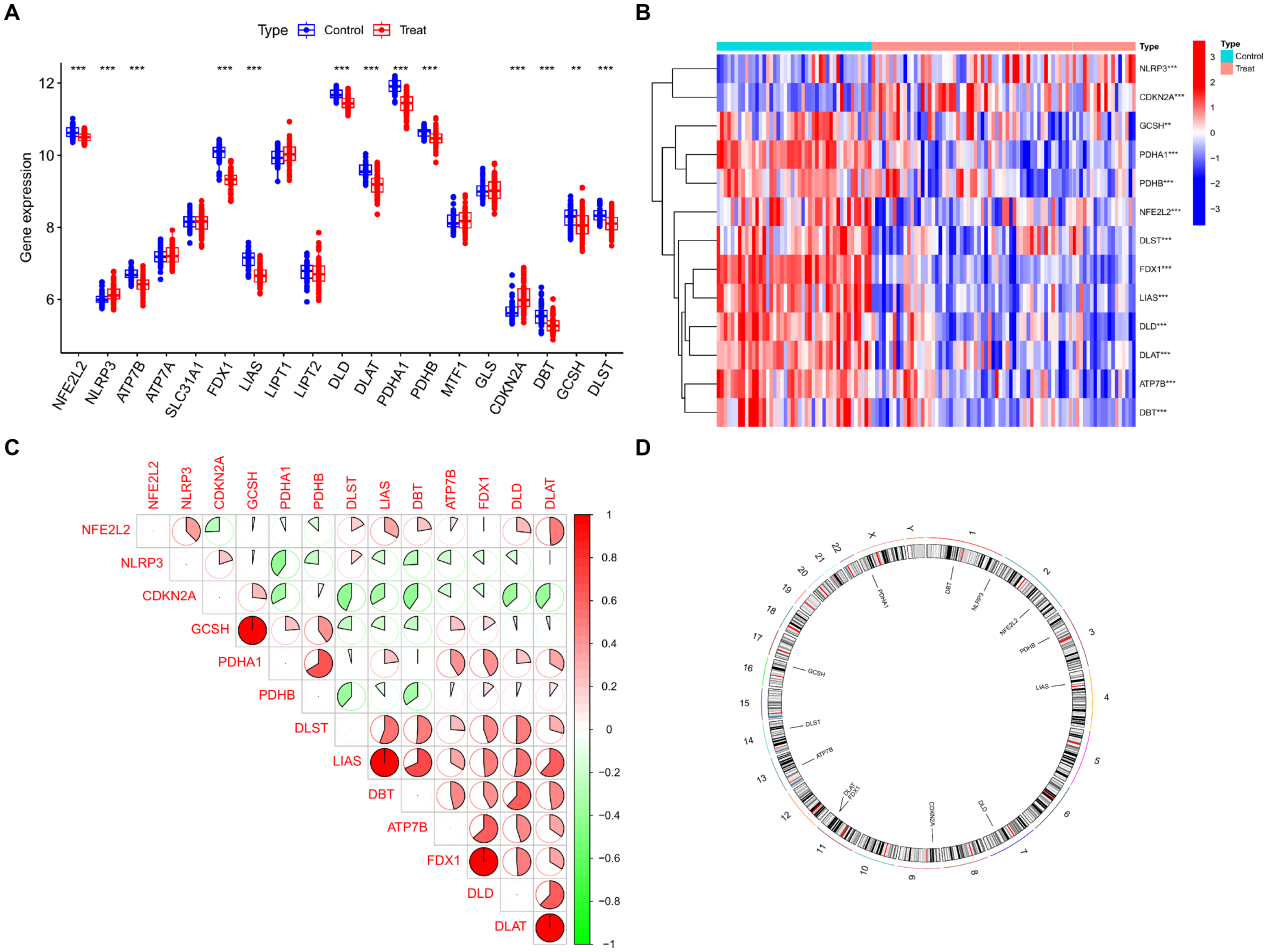
Identification of dysregulated CRGs in UC. **(A)** Boxplots showed the expression of 19 CRGs between UC and controls. **(B)** Correlation analysis of 13 differentially expressed CRGs. Blue and Red colors represent positive and negative correlations, respectively. **(C)** The correlation coefficients were marked with the area of the pie chart. **(D)** The location of 13 differentially expressed CRGs on chromosomes. ***p < 0.001, **p < 0.01, *p < 0.05.

Figure 2 displays the study’s flow chart. First, differential expression analysis on the GSE107499 dataset was conducted to identify 849 DEGs, comprising 309 up-regulated genes and 540 down-regulated genes (Figure 3A). The top 30 up-regulated and down-regulated genes were shown separately (Figure 3B). DEGs were also analyzed for GO and KEGG enrichment, and the findings demonstrated that the genes with differential expression were primarily engaged in immune-related pathways, such as leukocyte migration, leukocyte chemotaxis, cell chemotaxis in GO, and the cytokine-cytokine receptor interaction pathway in KEGG (Figure 3C). To elucidate the role of CRGs in the development of UC, the expression profiles of 19 CRGs were systematically evaluated using the GSE107499 dataset (Figure 4A). A total of 13 CRGs associated with UC were identified. In UC samples, the expression of NLRP3 and CDKN2A was significantly upregulated compared to normal samples. Conversely, the expression of NFE2L2, ATP7B, FDX1, LIAS, DLD, DLAT, PDHA1, PDHB, DBT, GCSH, and DLST was significantly decreased in UC samples. Differential analysis (Figure 4B) and correlation analysis (Figure 4C) were performed, followed by the visualization of chromosomal positions (Figure 4D).

### Evaluation of immune cell infiltration based on the CIBERSORT algorithm

**Figure 5:**
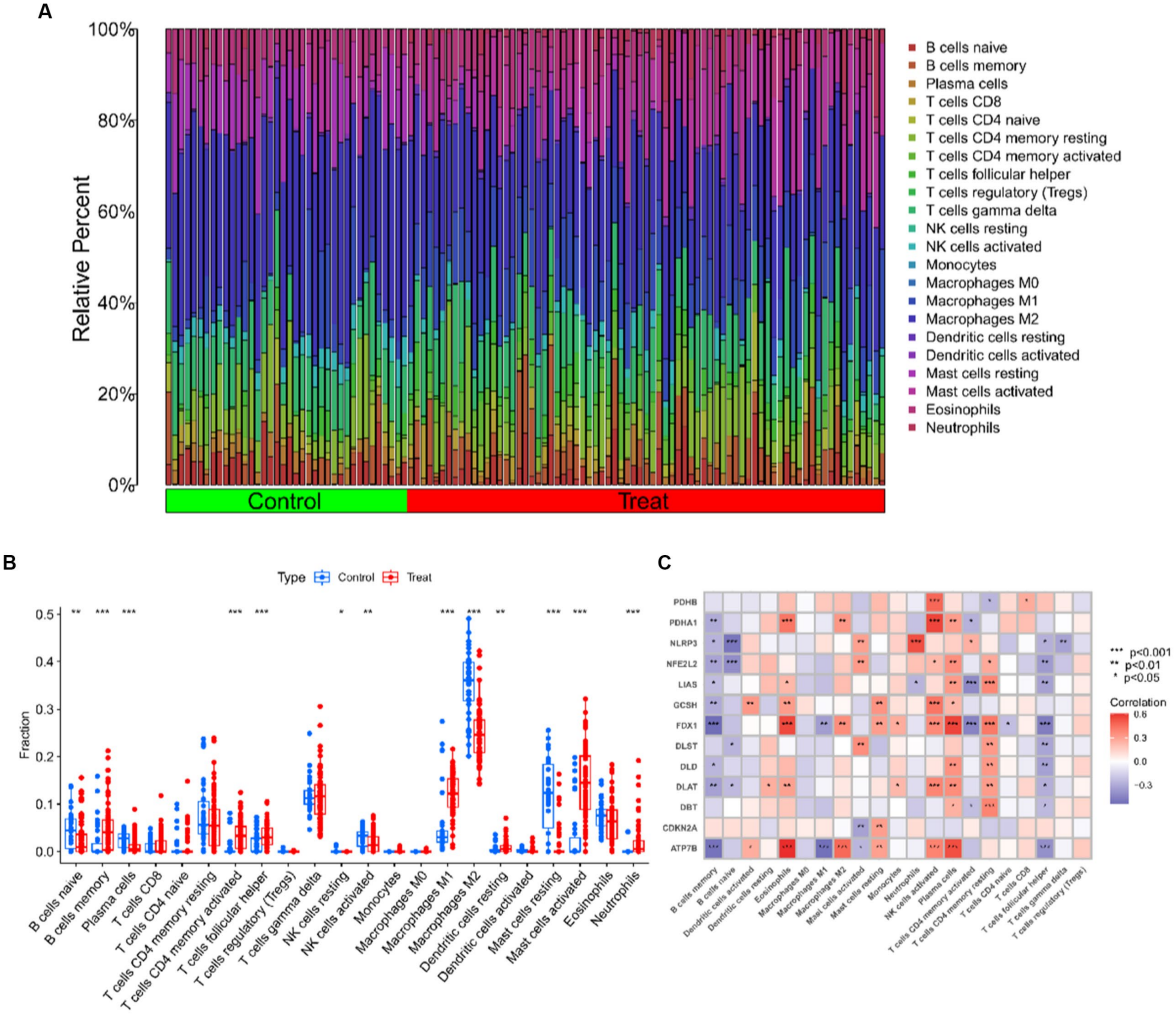
Immune cell infiltration analysis of UC and controls. **(A)** The relative abundances of 22 infiltrated immune cells between UC and controls. **(B)** Boxplots showed the differences in immune infiltrating between UC and controls. **(C)** Correlation analysis between 13 differentially expressed CRGs and infiltrated immune cells. ***p < 0.001, **p < 0.01, *p < 0.05.

The CIBERSORT algorithm was used to elucidate whether immune system differences existed between the UC and non-UC groups and to visualize differences in the proportions of the 22 infiltrating immune cell types, showing significant differences in B cells memory, T cells CD4 memory activated and Mast cells activated in UC patients (Figures 5A,B). The correlation analysis of the 13 CRGs with 22 immune cells showed that ATP7B showed a strong positive correlation with Eosinophils and Plasma cells, and a strong negative correlation with B cells memory and Macrophages M1. Eosinophils, T cells CD4 memory resting, and plasma cells all had a substantial positive connection with FDX1, as did T cells CD4 memory activated and T cells follicular helper (Figure 5C). These findings could imply that genes associated to cuproptosis control the infiltration of these immune cells, which in turn controls the development of UC.

### Unsupervised clustering of UC patients

**Figure 6:**
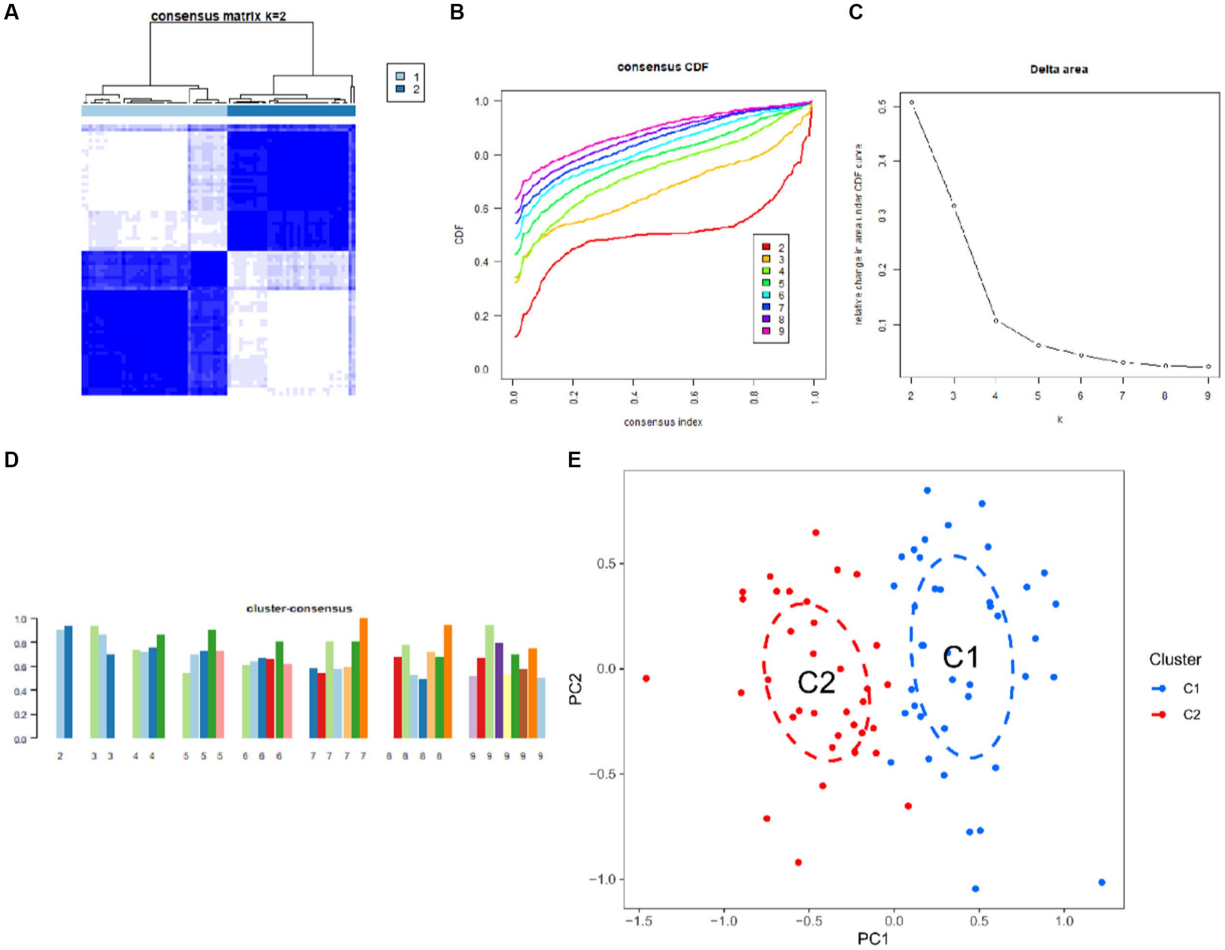
Identification of cuproptosis-related molecular clusters in UC. **(A)** Consensus clustering matrix when *k* = 2. **(B)** Representative cumulative distribution function (CDF) curves. **(C)** CDF delta area curves. **(D)** The score of consensus clustering. **(E)** PCA of two clusters.

**Figure 7:**
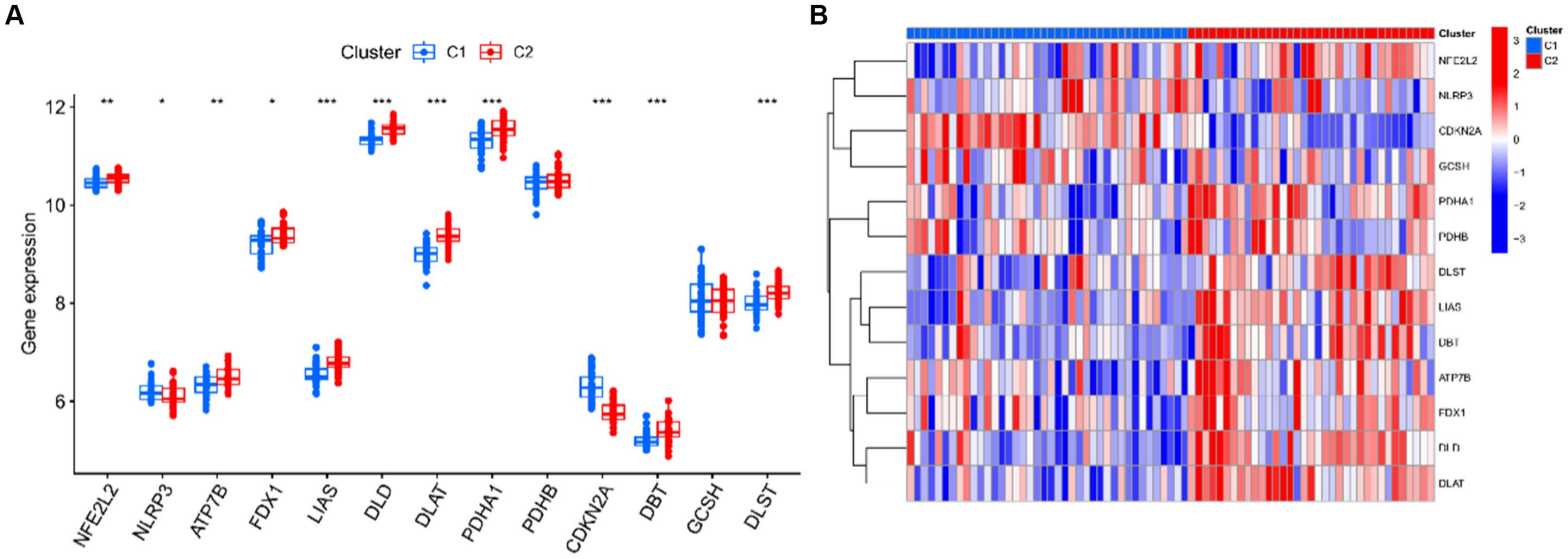
Identification differentially expressed CRGs of two clusters. **(A)** Boxplots showed the expression of 13 CRGs between two clusters. **(B)** Expression patterns of 13 CRGs between two clusters were presented in the heatmap. ***p < 0.001, **p < 0.01, *p < 0.05.

Seventy five UC samples were clustered using a trustworthy clustering technique based on the expression profiles of 19 CRGs in order to determine the CRGs expression patterns in UC samples. As soon as the k value was set to 2, the number of clusters was at its most stable (Figure 6A), and within a minimum range of 0.2–0.6 for the consensus indices, the CDF curve changed (Figure 6B). The difference between the two CDF curves is shown by the area under the CDF curve when *k* = 2–9 (Figure 6C). Additionally, only when *k* = 2 was the concordance score for each subtype larger than 0.85 (Figure 6D). In summary, we finally divided the 75 UC samples into two clusters, including cluster 1 (*n* = 40) and cluster 2 (*n* = 35). The results of a subsequent PCA analysis showed significant differences between these two clusters (Figure 6E). In addition, analysis of differences between the CRGs and the two clusters after clustering showed that there remained 11 CRGs (PDHA1, NLRP3, LIAS, ATP7B, FDX1, DLD, DLAT, NFE2L2, CDKN2A, DLST, and DBT) that were significantly different between the two clusters (Figures 7A,B).

### Immune cell infiltration analysis and GSVA analysis after clustering

**Figure 8:**
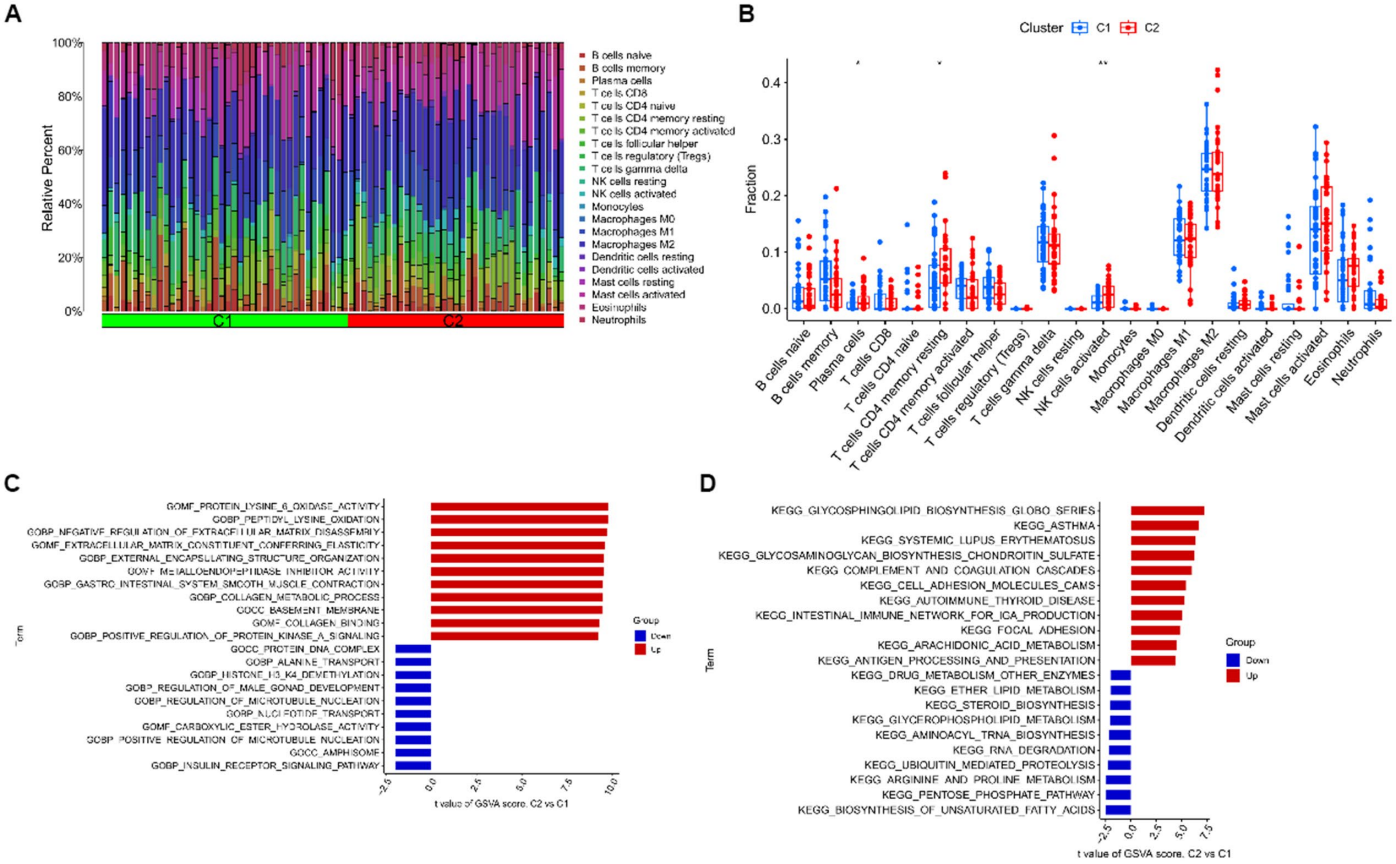
Immune cell infiltration analysis and GSVA analysis after clustering. **(A)** The relative abundances of 22 infiltrated immune cells between two clusters. **(B)** Boxplots showed the differences in immune infiltrating between two clusters. **(C)** Differences in biological functions between cluster 1 and cluster 2 samples ranked by *t*-value of GSVA method. **(D)** Differences in hallmark pathway activities between cluster 1 and cluster 2 samples ranked by *t*-value of GSVA method. ***p < 0.001, **p < 0.01, *p < 0.05.

For cluster 1 and cluster 2, we kept track of immune cell infiltration using the CIBERSORT algorithm. The findings demonstrated that plasma cells, CD4 memory resting T cells, and activated NK cells remained substantially different between the two clusters (Figures 8A,B). A GSVA analysis was then performed on both clusters. Functional enrichment results showed enhanced protein lysine 6 oxidase activity and negative regulation of extracellular matrix disassembly in cluster 1, while positive regulation of microtubule nucleation and carboxylic ester hydrolase activity were enhanced in cluster 2 (Figure 8C). In addition, pathway enrichment results showed that Glycosphingolipid biosynthesis, Glycosaminoglycan biosynthesis – chondroitin sulfate / dermatan sulfate and complement and coagulation cascades were enhanced in cluster 1, while Biosynthesis of unsaturated fatty acids, Pentose phosphate pathway and Arginine and proline metabolism were enhanced in cluster 2 (Figure 8D).

### Analysis of WGCNA for cluster 1 and cluster 2

**Figure 9:**
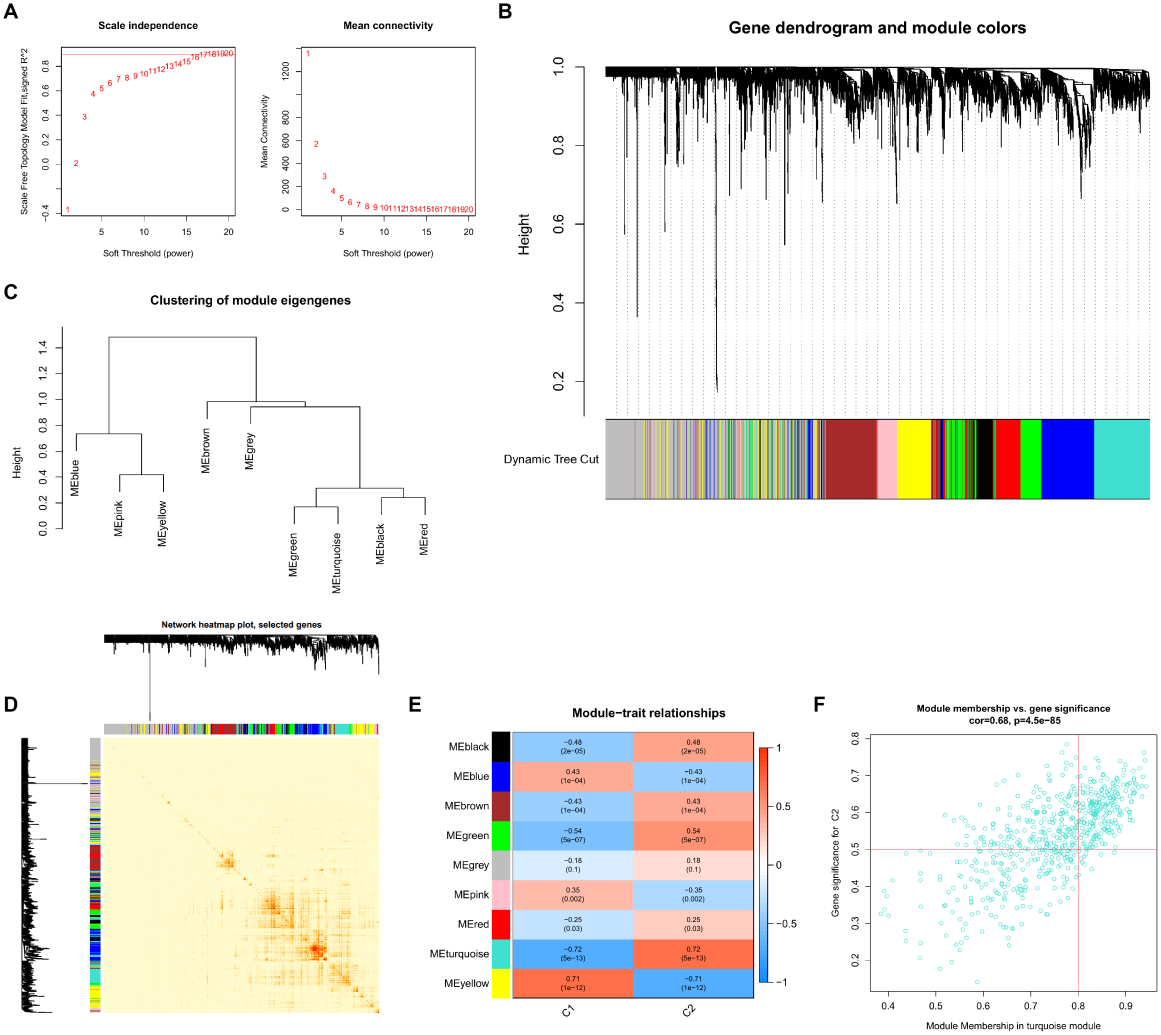
Co-expression network of differentially expressed genes between the two clusters. **(A)** The selection of soft threshold power. **(B)** Cluster tree dendrogram of co-expression modules. Different colors represent distinct co-expression modules. **(C)** Representative of clustering of module eigengenes. **(D)** Representative heatmap of the correlations among 11 modules. **(E)** Correlation analysis between module eigengenes and clinical status. Each row represents a module; each column represents a clinical status. **(F)** Scatter plot between module membership in turquoise module and the gene significance for cluster 2.

To locate key UC-associated gene modules, we built co-expression networks and modules for clusters 1 and 2 using the WGCNA algorithm. Co-expressed gene modules were discovered when the scale-free R2 was equal to 0.9 and the soft threshold was set at 16 (Figure 9A). The dynamic cutting method produced eight different colored co-expression modules, and a heat map of the topological overlap matrix is also displayed (Figures 9B–D). These eight color modules’ genes were then sequentially used to examine the similarity and proximity of the co-expression of the module’s clinical characteristics (cluster 1 and cluster 2). The turquoise module, which contains 283 genes, exhibited the highest correlation with cluster 2 (Figure 9E). Furthermore, significant relationships between the turquoise module genes and the chosen module genes were shown by correlation analysis (Figure 9F).

### Machine learning predictive models

**Figure 10:**
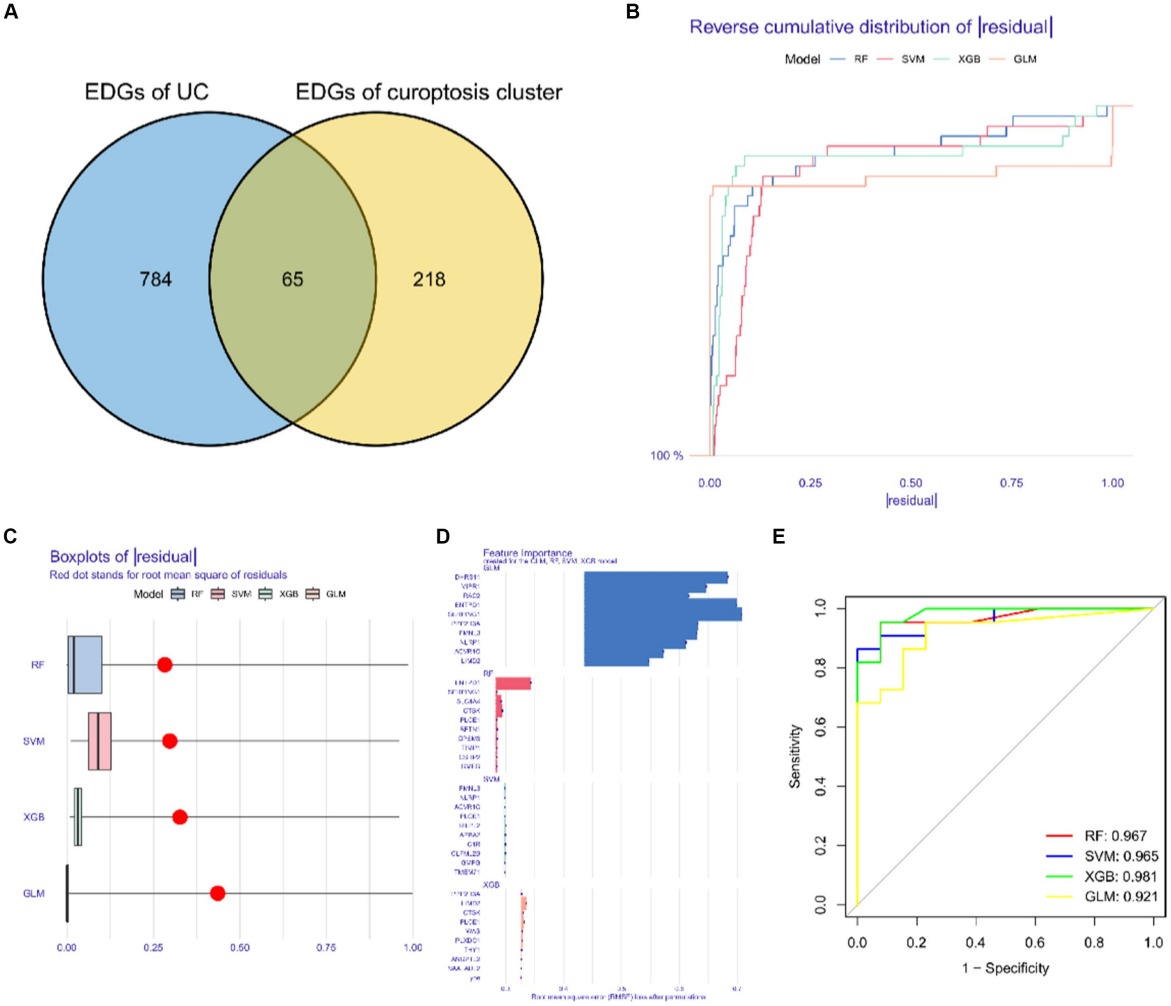
Construction and evaluation of XGB, SVM, RF and GLM machine models. **(A)** Common differentially expressed genes. **(B)** Cumulative residual distribution of each machine learning model. **(C)** Boxplots showed the residuals of each machine learning model. Red dot represented the root mean square of residuals. **(D)** The important features in XGB, SVM, RF and GLM machine models. **(E)** ROC analysis of four machine learning models based on 5-fold cross-validation in the testing cohort.

We identified a total of 65 intersecting genes for DEGs of UC and Cluster 2 (Figure 10A). To further identify these intersecting genes as having high diagnostic value, based on the 65 cluster-specific DEGs’ expression characteristics in the UC training cohort, we developed four well-established machine learning models: XGB, SVM, RF, and GLM. The “DALEX” package (version 2.4.0) was used to examine the four models and to show the residual distributions for each model in the test set. The machine learning models for XGB and RF showed very little residuals (Figures 10B,C). Following that, based on root mean square error, the top 15 significant feature variables for each model were ranked (Figure 10D). In addition, on the basis of 5-fold cross-validation, by developing ROC curves, in the test set, we evaluated the four machine learning algorithms’ discriminative performance. The AUC of XGB was 0.981, the AUC of RF was 0.967, the AUC of SVM was 0.965, and the AUC of GLM was 0.921 (Figure 10E). In summary, the XGB model had the highest accuracy. The top 5 of these genes were PLXDC1, WAS, CTSK, PLCE1, and LIMD2.

### Nomogram model construction and independent validation analysis

**Figure 11:**
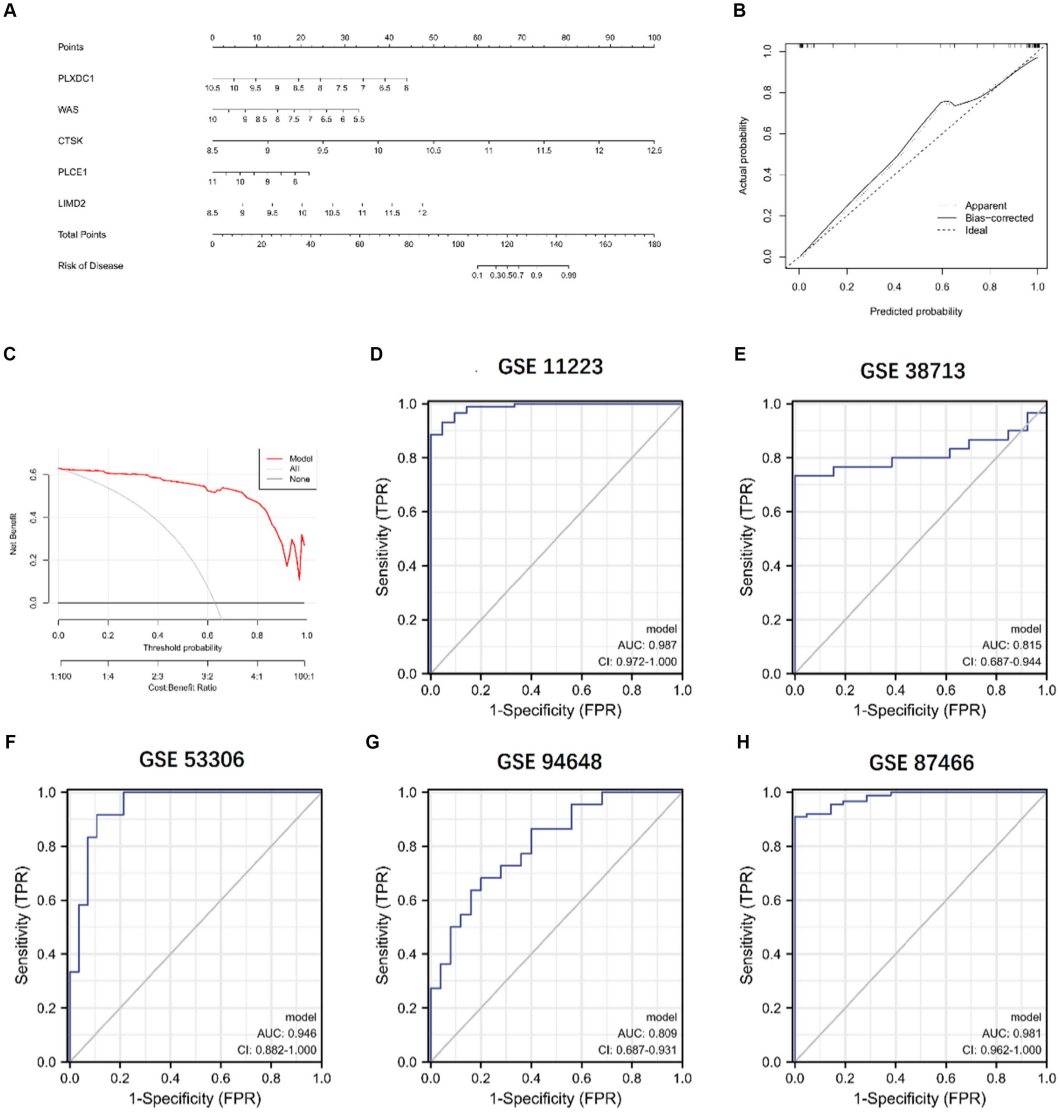
Validation of the 5-gene-based XGB model. **(A)** Construction of a nomogram. **(B,C)** Construction of calibration curve **(B)** and DCA **(C)** for assessing the predictive efficiency of the nomogram model. **(D–H)** ROC analysis of the 5-gene-based XGB model based on 5-fold cross-validation in GSE11223 **(D)**, GSE38713 **(E)**, GSE53306 **(F)**, GSE94648 **(G)**, and GSE87466 **(H)**.

We constructed a column line plot to evaluate the predictive accuracy of the XGB model (Figure 11A), and the scores for each gene were summed to obtain a total score, which corresponded to the disease risk value. To assess the precision of the column plots, calibration curves and DCA curves were created, which showed high accuracy (Figures 11B,C). The AUC of the five independent validation datasets were GSE11223 (AUC = 0.987), GSE38713 (AUC = 0.815), GSE53306 (AUC = 0.946), GSE94648 (AUC = 0.809), and GSE87466 (AUC = 0.981), suggesting high accuracy (Figures 11D–H).

## Discussion

UC is characterized by recurrent episodes of chronic inflammation of the colon and places a great physical and financial burden on patients (37). The mechanisms of disease development are not yet clear (38). The main drugs used for treatment are mesalamine and steroids. A new type of cell death known as cuproptosis has recently been described. It is characterized by an excessive amount of intracellular copper accumulation, which causes an accumulation of mitochondrial lipid acylated proteins and the destabilization of Fe-S cluster proteins, which ultimately causes cell death (9, 39). However, its particular processes and its regulatory function in different disorders have not been fully analyzed. We thus made an effort to clarify the precise function of CRGs in UC samples and their immunological milieu in order to offer some theoretical support for relevant investigations to come. In addition, CRGs can be used to further predict the subtypes of UC, thus providing more accurate and individualized treatment.

In this study, we have for the first time comprehensively analyzed the expression profiles of CRGs in normal and UC samples. The expression of CRGs varied dramatically between the two groups, pointing to a crucial role for CRGs in the development of UC. ATP7B encodes a copper-transporting ATPase, which maintains copper balance in the body (11). FDX1 participates in intracellular electron transfer processes, playing a vital role in various metabolic pathways, including iron–sulfur cluster biosynthesis and heme synthesis (40). Additionally, DLD and DLAT encode proteins involved in the pyruvate dehydrogenase complex (PDH), crucial for converting pyruvate to acetyl-CoA. These genes work in conjunction with PDHA1 and PDHB, which encode subunits of the PDH complex. Deficiencies in the PDH complex can result from mutations in these genes (41–43). Furthermore, DBT is responsible for encoding a protein component of the branched-chain alpha-keto acid dehydrogenase complex, while DLST encodes a protein involved in the alpha-ketoglutarate dehydrogenase complex, contributing to the tricarboxylic acid (TCA) cycle and energy metabolism (41). GCSH is associated with the glycine cleavage system (44). LIAS, on the other hand, encodes lipoic acid synthetase, a necessary cofactor for several enzyme complexes involved in energy metabolism (45). Additionally, NFE2L2 plays a crucial role in cellular defense against oxidative stress, while NLRP3 primarily functions in innate immunity and inflammatory responses. Activation of the NLRP3 inflammasome can trigger the release of pro-inflammatory cytokines (46, 47). Finally, CDKN2A acts as a tumor suppressor gene, regulating cell cycle progression and participating in cellular senescence and apoptosis (48). Additionally, these studies have revealed that the identified CRGs play significant roles in diverse biological pathways beyond their association with cuproptosis. The examination of immune cells that infiltrated UC and the relationship between these cells and CRGs were then carried out. The findings demonstrated a substantial difference in immune cell abundance between normal and UC samples, with UC samples exhibiting considerably greater immune infiltration levels of memory B cells, T cells with CD4 memory activation, and activated mast cells than the normal group. In addition, we used WGCNA analysis to identify two subtype clusters based on the differential expression of CRGs in 75 UC samples. With enhanced immune fractions and relatively high levels of immune infiltration, the pathway of cluster 2 was mostly enriched in the intestinal immune network for the generation of immunoglobulin A.

A number of machine learning algorithms have been widely used in recent years to forecast the incidence of UC (49), and these researches have shown that multifactorial analysis is more accurate than univariate analysis, with lower error rates. In this research, four machine learning models (XGB, SVM, RF, and GLM) were created, and their prediction capabilities were examined in order to develop the best prediction model based on XGB, which presented the highest prediction accuracy (AUC = 0.981) among the four models, indicating that the XGB-based machine learning model has a satisfying outcome. Subsequently, we selected the first five important variables (differentially expressed genes), namely PLXDC1, WAS, CTSK, PLCE1, and LIMD2, to construct an UC-XGB model based on these five genes. Currently, various studies have reported that XGB models are widely used and perform well in several disease areas, such as predicting bone metastases in patients with prostate cancer and lymph node metastases in patients with melanoma and osteosarcoma (50–52). The UC-XGB model constructed in this study can minimize error, maximize the performance of the model, and effectively prevent overfitting. Compared with the traditional linear model, the UC-XGB model, although increasing the computational effort, can get rid of the constraints imposed by the traditional linear model due to the fixed coefficients of each variable and can utilize the semantic information more flexibly and exploit the underlying patterns more fully. Therefore, the UC-XGB model constructed based on PLXDC1, WAS, CTSK, PLCE1, and LIMD2 in this study has excellent performance. The protein PLXDC1 (Plexin Domain Containing 1) was first discovered to be significantly expressed in the endothelium of human tumor vessels (53). PLXDC1 is significantly expressed in tumor endothelial cells and has been demonstrated to be involved in tumor angiogenesis (54, 55). Abnormalities in PLXDC1 have been reported to be closely associated with tumor disease, and PLXDC1 has been demonstrated to be a biomarker for immune evasion and a poor prognosis in gastric cancer (56). The WAS gene product is a cytoplasmic protein that is characteristically expressed only in hematopoietic cells (57). Wiskott-Aldrich syndrome, a condition marked by immunological dysregulation, can be brought on by mutations in the WAS gene (58). Cathepsin K is a lysosomal cysteine protease that is involved in bone remodeling and resorption (59). It is a protein-coding gene. This gene is closely linked to the emergence of a number of disorders in addition to being engaged in the regulation of the body’s normal physiological processes. CTSK has been found to be expressed in a variety of cells, such as heart, colon, small intestine and other tissues and osteoblasts, among others (60, 61). Phospholipase C Epsilon 1, also known as PLCE1, catalyzes the hydrolysis of the second messenger phosphatidylinositol 4,5-bisphosphate (PIP2) to produce two crucial second messengers that control the intracellular interactive signaling network (62, 63). In addition, PLCE1 regulates complex signaling pathways and affects the development of a variety of tumors (64). The protein-kinase ILK is activated by LIMD2 (LIM Domain Containing 2), which controls cell motility (65). LIM structural domains have been shown to be key molecules in various human cancers, and it has recently been established that LIMD2, a member of the LIMD family, is linked to the emergence and spread of human malignancies (66, 67). Furthermore, by focusing on miR-34a, LIMD2 promotes the growth and invasive migration of non-small cell lung cancer (68). At last, a reliable predictor for determining UC subtypes and pathological outcomes in UC patients is the five-gene-based XGB model.

There are also several limitations to the present study. First of all, there was no clinical or experimental evaluation to evaluate the expression levels of CRGs; instead, our current work was conducted based on a thorough bioinformatics analysis. Secondly, in order to clarify the reliability of CRGs and further investigate the potential relationship between CRGs and immunological responses, additional UC samples are required. Finally, the five genes mentioned above are relatively few in the relevant studies of UC, and their functions and values need further validation. Therefore, we will conduct comprehensive functional trials in the future to elucidate the complex mechanisms of action of the five CRGs and will recruit a wide range of clinical patients to further validate the clinical value of our CRGs.

## Conclusion

In conclusion, we first identified CRGs and immune correlates with differential expression in normal and UC patient samples. Based on the expression of CRGs, UC patient samples were divided into two clusters and important immune-related differences between UC patients with different CRGs clusters were elucidated. Subsequently, the WGCNA algorithm was used to identify DEGs with enriched biological functions and pathways in both clusters. Finally, we constructed an XGB machine learning model based on five CRGs (PLXDC1, WAS, CTSK, PLCE1, and LIMD2). Nomograms, calibration curves, DCA, and independent external datasets all validated the accuracy of the model. Our study reveals the function of CRGs in UC for the first time, and we hope that the CRGs elucidated in this study can provide important inspiration for subsequent studies on the functional mechanisms of UC and guide clinicians to make more individualized and precise clinical treatment plans.

## Data availability statement

The datasets presented in this study can be found in online repositories. The names of the repository/repositories and accession number(s) can be found in the article/Supplementary material.

## Ethics statement

Written informed consent was obtained from the individual(s) for the publication of any potentially identifiable images or data included in this article.

## Author contributions

ZW and XW: conception and design. XL: administrative support. XW and XL: provision of study materials or patients. JY: collection and assembly of data. YZ and YWe: data analysis and interpretation. All authors contributed to the article and approved the submitted version.

## Conflict of interest

The authors declare that the research was conducted in the absence of any commercial or financial relationships that could be construed as a potential conflict of interest.

## Publisher’s note

All claims expressed in this article are solely those of the authors and do not necessarily represent those of their affiliated organizations, or those of the publisher, the editors and the reviewers. Any product that may be evaluated in this article, or claim that may be made by its manufacturer, is not guaranteed or endorsed by the publisher.
